# Major Stress-Related Symptoms During the Lockdown: A Study by the Italian Society of Psychophysiology and Cognitive Neuroscience

**DOI:** 10.3389/fpubh.2021.636089

**Published:** 2021-03-26

**Authors:** Sara Invitto, Daniele Romano, Francesca Garbarini, Valentina Bruno, Cosimo Urgesi, Giuseppe Curcio, Alberto Grasso, Maria Concetta Pellicciari, Giacomo Koch, Viviana Betti, Mirta Fiorio, Emiliano Ricciardi, Marina de Tommaso, Massimiliano Valeriani

**Affiliations:** ^1^INSPIRE LAB - Laboratory of Cognitive and Psychophysiological Olfactory Processes, Department of Biological and Environmental Sciences and Technologies, University of Salento, Lecce, Italy; ^2^Department of Psychology, University of Milano-Bicocca, Milan, Italy; ^3^Manibus Lab, Department of Psychology, University of Torino, Torino, Italy; ^4^Department of Languages and Literatures, Communication, Education, and Society, University of Udine, Udine, Italy; ^5^Dipartimento di Scienze Cliniche Applicate e Biotecnologiche, Università Degli Studi Dell'Aquila, L'Aquila, Italy; ^6^UniCamillus, Saint Camillus International University of Health Science, Rome, Italy; ^7^Fondazione Santa Lucia IRCCS, Rome, Italy; ^8^Dipartimento di Neuroscienze e Riabilitazione, Università di Ferrara, Ferrara, Italy; ^9^Department of Psychology, Sapienza University of Rome, Rome, Italy; ^10^Department of Neurosciences, Biomedicine and Movement Sciences, University of Verona, Verona, Italy; ^11^IMT School for Advanced Studies, Lucca, Italy; ^12^Department of Basic Medical Science, Neuroscience and Sense Organs, University of Bari Aldo Moro, Bari, Italy; ^13^Neurology Unit, Bambino Gesù Hospital, Rome, Italy

**Keywords:** Covid-19, pain, sleep habits, olfactory perception, lockdown, stress, anxiety symptoms

## Abstract

The clinical effects of the Covid-19 pandemic are now the subject of numerous studies worldwide. But what are the effects of the quarantine imposed by the states that implemented the measures of lockdown? The present research aims to explore, in a preliminary way, the major stress-related symptoms during the lockdown, due to Covid-19, in the Italian population. Subjects were asked to fill out a survey, that traced a line identifying the most relevant psychophysiological symptoms that took into account factors such as perceived stress, body perception, perceived pain, quality of sleep, perceptive variations (i.e., olfactory, gustatory, visual, acoustic, and haptic perception). A network approach formulating a hypothesis-generating exploratory analysis was adopted. Main results of the network analysis showed that the beliefs of having had the Covid-19 was related to individual variables (i.e., gender, working in presence, sleep quality, anxiety symptoms), while the familiarity of Covid-19 disease was related to contextual factors (e.g., number of recorded cases in the Region, working in presence). The self-perception of olfactory and perceptive alterations highlighted a great sensorial cross-modality, additionally, the olfactory impairment was related to the belief of having had the Covid-19. Compared to general network data, BAI, perceived stress, anxiety and chronic pain were in relation to daily sleep disturbance. Main study's results show how the management of the Covid-19 stressful representation, in its cognitive aspects, can modulate the psychophysiological responses.

## Introduction

The present work aims to describe the potential long-term psychophysiological effects of the restrictions applied by the Institutions during the Coronavirus pandemic, a world emergency condition that forced the population to go into lockdown or/and quarantine.

The term “quarantine” and the term “lockdown” have become commonly used today. The term quarantine indicates a period of safety, needed to limit the infections, that is imposed on one or more persons who have or may have contracted a highly contagious syndrome that is needed to limit infections. Quarantine differs from isolation which, on the other hand, coincides with the separation of infected individuals from the rest of potentially healthy individuals (1). The term lockdown instead represents a strict limitation of relational, work and social activities (2).

During the Covid-19 pandemic, the governments of many countries imposed a long period of lockdown or quarantine on citizens in order to monitor the epidemic and keep potential infections under control: but there is talk of a measure of social containment to which no one could be prepared for, which upset daily habits and severely impacted psycho-physical well-being (1, 3, 4). The negative psychological effects that the lockdown left on the world population are now documented and space is further opened for new studies that will evaluate the effects over time.

In fact, during the quarantine period there are numerous stressors that, according to the literature, contribute to making us experiencing the period of social distancing in an even more difficult way. In this regard, it was found that the longer the duration of the quarantine, the more likely it is to develop feelings of anger, symptoms of post-traumatic stress disorder and phobic avoidance behaviors (5). Especially, there seems to be fear of developing symptoms of the disease and infecting others (6). The loss of the job, the daily routine and the cancellation of social contact are then often indicated as causes of negative feelings, such as boredom, demoralization, a sense of loneliness and isolation from the rest of the world. It also emerges that the fear of not having supplies available for subsistence, such as food or drugs, is a source of additional stress, which causes anxiety, anger, and frustration in people (5).

The data collected from previous pandemics suggest the probability that during the period of social distancing, phobic or obsessive disorders may develop that persist long after the end of the epidemic. Research conducted on individuals who had been quarantined due to possible contact with the SARS virus found that after the emergency ended, 54% of people who had been placed in isolation avoided coughing or sneezing, 26% avoided closed and crowded places and 21% avoided all public spaces (7). A related long-term study, carried out after the quarantine period, highlighted the presence of behavioral changes aimed at reducing the hypothetical risk of contagion, such as compulsive hand washing and avoidance of crowded places (8). In addition, an analysis conducted on hospital staff who had come into contact with SARS patients, found that acute stress symptoms were reported after the end of the quarantine period, such as severe anxiety, irritability, insomnia, poor concentration and decreased productivity work (9).

In light of this recent literature, this political-health condition could be considered a chronic stressor for the body. In fact, the effects of chronic stress, in this particular Covid-19 pandemic period, are not due exclusively to health aspects, but to political and social indications linked to constraints not only on lifestyle and on the possibilities of movement and social interaction, but also to economic conditions directly caused by the lock down (10, 11). It is well-known that psychological states can influence physiological responses, and more in general, physical health. For example, states of anxiety and stress are accompanied by physiological changes which can be regarded as high arousal (12), with augmented skin conductance response, increased startle reflex and greater pupil dilation (13). The levels of cortisol, the hormone involved in the fight or flight response to potential threats, are characterized by a relatively rapid increase followed by a progressive decline in adaptive responses to stress, while on the contrary, flatter reactivity and recovery is a maladaptive response often called blunted reactivity (14). In general, feelings of anxiety and stress are accompanied by physical signs and symptoms such as palpitations, a sense of constriction in the chest, tightness in the throat, difficulty in breathing, epigastric discomfort or pain, dizziness and weakness in the legs, dryness of the mouth, sweating, vomiting, tremor, running in panic and sudden micturition (15). In the last months, it has been stressed that prolonged home confinement during a disease outbreak may affect people's physical and mental health (16, 17). This can happen by reducing the level of physical activity and the exposure to daylight, and by increasing the level of stress due to social isolation and the impossibility to engage in satisfying activities. The direct effects of these changes are both the disruption of night-time sleep (18) and the increase of the risk of mental health problems (1, 19): usually these effects are very interconnected to each other and might be seen as the potential first steps toward more severe symptomatology, such as post-traumatic stress disorder (PTSD). In fact, increased stress and greater impact of depression and anxiety symptomatology have been reported all around the world as a consequence of the Covid-19 pandemic (20, 21). Recent results from a study carried out in Italy (6) reported that the recent pandemic appeared to be a risk factor for sleep disorders and psychological diseases also in the Italian population, as previously seen in China. Another study (22) showed that during home confinement, sleep timing markedly changed, with people going to bed and waking up later (spending more time in bed) with a paradoxical reduction of sleep quality. The authors claimed that the increase in sleep difficulties was stronger for people with a higher level of depression, anxiety and stress symptomatology. Such individual and gender differences have been confirmed as very relevant, highlighting a different time course of sleep and mental health between genders during the home confinement period, with women showing greater long-term resilience during the lockdown and males as the most vulnerable to the extension of the restraining measures (23).

Furthermore, changes in the amount of physical activity could be considered as a possible factor contributing to changes in perceived stress during the quarantine. Accumulating evidence suggests that perceived stress is inversely related to the amount of physical activity and positively associated with sedentary time, especially in the young population (24). In this frame of reference, it could be hypothesized that reduced physical activity induced by the lockdown could have an impact on perceived stress (25, 26). However, a study in the Italian population showed that physical activity levels were not reduced during lockdown, but rather slightly increased (27), thus ruling out a role of this factor as potential stressor in our country.

This highly stressful condition, together with the information passed on by the media, on the related Covid-19 symptoms, can strongly change, therefore, not only the condition of perceived stress and the levels of anxiety, but also the body perception, sensory, perceptual and psychophysiological parameters (including nociception). In fact, even purely sensory parameters can vary significantly when associated with stressful situations (28–30). Starting from this literature background, the aim of this research was to explore, in a preliminary way, the major stress-related symptoms during the lockdown, due to Covid-19, in the Italian population. To investigate these psychophysiological variations, we observed, through an exploratory analysis, the networks between heterogeneous aspects. In particular, we identified two main networks. The first network (Network A) aimed at connecting individual differences (e.g., gender, sleep quality, anxiety symptoms), social (e.g., education level, working in presence) and contextual variables (e.g., number of cases recorded in the region) to the belief of having had the Covid-19, or that a family member had it. The second network (Network B) aimed at exploring how belief of having had the Covid-19 was related to perceived sensory modulations and changes (e.g., altered olfactory perception, vision, or taste). The idea of using this network model arose from the fact that we did not have a network baseline among these variables, and we did not have possible control model od Covid-19. The only possibility was to link relational pathways to Covid-19 sensory and psychophysiological aspects, assessed in a pandemic condition, and correlated to the perceived stress.

## Methods

### Study Population

In total, 2.992 participants (Mean age = 39.36; sd = 15.06; 75.5%women) were engaged for compilation of the online survey. The survey was available from March to May 2020, during the first Italian lock down. Subjects were recruited through online announcements on the social and official websites of the Society of psychophysiology and cognitive neuroscience (i.e., www.sipf.it; SIPF Twitter, SIPF Facebook) and through the advice on the link of the SIPF members of each research unit included in this study. The participants were not recruited individually but through the online announcements, so we could not quantify the number of people who read the advice but did not participate in the research. The research participation was voluntary, anonymous and did not include study exclusion criteria. Any subject reported a psychiatric diagnosis. An information sheet preceded the survey, including information on what the research was about, the reason for conducting the study, how the data would be used, how privacy of data would be maintained, the benefits and risks of taking part in the survey, along with contact details for further information. Ethical approval was obtained from the local Ethics Committee of the University of Torino (prot. n. 147807, 30.03.2020).

### Survey

The Survey had to be filled out anonymously *via* Google Form and was divided into two sections: the first one included personal data (e.g., gender, age, etc.), socio-economic (e.g., smart working, work in presence; no work, etc.), geographic, and medical information (e.g., chronic diseases, Covid-19 disease; the belief to have had Covid-19; familiars affected by Covid-19, etc.); the second section included a series of behavioral questionnaires aimed at investigating the main psychophysiological, emotional and perceptive functioning stress related. In particular, were assessed the following variables: the perceived stress with the Perceived Stress Scale (PSS) (31), the anxiety with The Beck Anxiety Inventory (BAI) (32, 33), the body perception with the Body Perception Questionnaire BPQ (34), the chronic pain with the Von Korff scale (35), the sleep disorders with the Pittsburgh Sleep Quality Index (PSQI) (36) using a revised scoring system has been developed based on a 3-factor model based on perceived sleep quality (F1), sleep efficiency (F2) and daily disturbances (F3) (36, 37); finally the Survey proposed a self-assessment scale of one's perceptual and sensorial sensitivity (sight, hearing, taste, smell, touch), and an item on any perceived variation in eating habits. For the analysis of the psychophysiological data, the geographic-contextual variables (i.e., number of Covid-19 positive cases in the Region, number of Covid-19 deaths in the Region) was also considered.

### Descriptive Data

The geographic distribution of the participants was quite heterogeneous: Abruzzo (1%), Basilicata (1%), Calabria (1.5%), Campania (2.3%), Emilia Romagna (2%), Friuli Venezia Giulia (0.4%), Lazio (22.9%), Liguria (3.2%), Lombardia (13.08%), Marche (0.47%), Molise (0.17%), Piemonte (11.8%), Puglia (19.56%), Sardegna (6.47%), Sicilia (3.13%), Toscana (9.4%), Trentino Alto Adige (0.17%), Umbria (0.9%), Valle d'Aosta (0.07%), Veneto (3.24%). During the survey availability all the Italian regions was in lockdown. Only 16 (0.53%) subjects declared they had Covid-19, while 259 (8.7%) subjects said they believed they had Covid-19, 95 (3.2%) subjects declared to have a familiar affected by Covid-19 and 1072 (35.8%) subjects declared to have friends with Covid-19. The education qualification was represented as follow: junior high school diploma 6.9%, high school diploma 37.8%, master's degree 35.9%, post-graduate training 19.4%. Education level was not added to the Network analysis because it is an ordinal variable that can't be modeled appropriately with Gaussian Graphical Model (GGM) (38).

#### Network Analysis

A network is composed of a set of elements named nodes (i.e., the variables) and their connections named edges (i.e., the relationship). It offers the opportunity of analyzing multiple nodes and the complexity of their edges, giving back a manageable output. For example, networks have been used to model either personality and attitudes in healthy and pathological situations (39–44), or neuropsychological performances in adults (45).

The edges in networks assessing psychological phenomena can be estimated with different methods according to the different types of data.

#### Network A

When a mix of dichotomous and continuous variables are included the network, like in our Network A, is typically estimated through the GGM (38). By adopting a GGM, edges indicate regularized partial correlations.

The GGM network estimation employs the “least absolute shrinkage and selection operator” (LASSO); (46) algorithm as the regularization parameter (44). The LASSO reduces small correlations to zero (47, 48), by doing so, it reduces the overfitting and limits the finding of false-positive edges, returning a conservative, replicable and interpretable network (43).

The Extended Bayesian Information Criterion was used to select the LASSO value, a method regulated by a parameter γ, that was set at 0.25, a standard value for the psychological literature (49).

Because of the regularization parameter, this method may have a low sensitivity (i.e., not all real edges are detected) but it has a high specificity (i.e., few false positives) (50).

The strength centrality index quantifies the importance of a node according to the number of its neighbors (i.e., edges connected) and the strength of its connections by summing the absolute value of each edge passing through a node (51).

#### Network B

When only dichotomous variables are included in the network, a better way to estimate the edges is by using the IsingFit model (50). The Ising algorithm uses LASSO-regularized logistic regressions and it was developed to deal with binary data (52).

The IsingFit network estimates the edges as follows: (a) a series of logistic regressions is run where at different steps one variable is the dependent variable, which is regressed on all the others. (b) each variable is put in the dependent variable spot iteratively; this results in having two parameters of association for every couple of nodes. (c) the LASSO is used as a parameter of regularization, reducing to 0 those coefficients that have little predictive value. (d) the final edge estimation is calculated by using the mean of the two parameters for each edge.

The networks analyses for A and B were performed using the JASP software [JASP Team (2020), Version 0.14].

#### How to Interpret a Network

Edges have no causal meaning *per se*. Each edge indicates conditional dependence between two variables net of the other variables. Basically, it says if two variables are associated after taking out all the variance explained by the other variables.

Thus, when an edge connects two nodes, it means that they are directly associated. The edge expresses the unmediated relation of the two nodes, the variance uniquely shared by the two. When two nodes are disconnected, it means that there is no variance uniquely shared by the two variables, thus any possible simple correlation observed between the two nodes can be explained by the covariance with the other nodes.

A network can also be seen as a predictive model, in which the neighbors of each node are its predictors. Thus, a central node is also a node highly predictable given the others. This means that a node with high strength centrality is more predictable given the others (51).

## Results

### Network A

**Figure 1:**
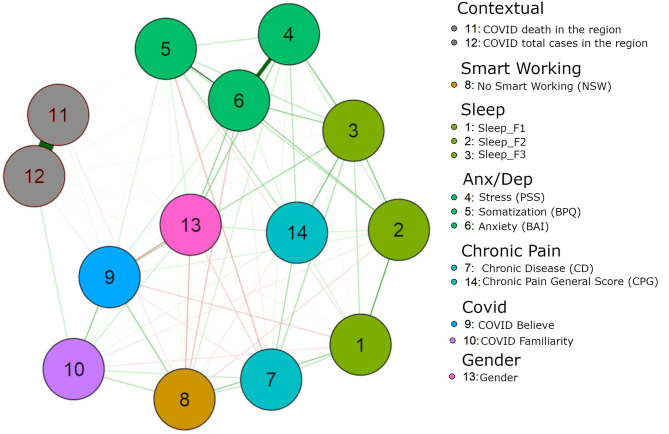
Green lines indicate positive associations. Red lines indicate negative associations. Values are regularized partial correlations. The nodes indicate the variables as follow: 1 Sleep_F1; 2 Sleep_F2; 3 Sleep_F3; 4 PSS (Perceived Stress Scale); 5 BPQ (Body Perception Questionnaire); 6 BAI (Beck Anxiety Inventory); 7 CD (Chronic Disease); 8 NSW (Not Smart Working); 9 Covid B (Covid Believe); 10 Covid F (Covid Familiarity); 11 Covid Death in the Region of the responder; 12 Covid Tot cases in the Region of the responder; 13 Gender; 14 CPG (Chronic Pain General Score).

Figure 1 represents the best network estimated from the data. The exact value of all edges is reported in Table 1, which also shows the strength centrality index on the diagonal.

**Table 1:** The matrix reports the network weights, which corresponds to regularized partial correlations.

**Variable**	**Sleep_F1**	**Sleep_F2**	**Sleep_F3**	**PSS**	**BPQ**	**BAI**	**CD**	**NSW**	**Covid B**	**Covid F**	**Covid Death**	**Covid Tot**	**Gender**	**CPG**
Sleep_F1	0.813	0.287	0.087	0.01	−0.038	0	0.079	0.154	−0.061	−0.031	0	−0.011	−0.009	0.043
Sleep_F2	0.287	0.913	0.183	0.05	0.09	0.14	0	−0.041	0.037	−0.024	1.655*e*−4	0	−0.009	0.048
Sleep_F3	0.087	0.183	1.174	0.19	0.085	0.147	0.11	−0.025	0.126	0.007	0	−0.009	0.009	0.2
PSS	0.013	0.052	0.186	1.16	0.071	0.446	0.025	0.052	0.048	0.017	0	−0.01	0.146	0.095
BPQ	−0.038	0.09	0.085	0.07	0.911	0.31	−0.095	0.018	0.041	0.01	0.003	3.443*e*−4	0.045	0.106
BAI	0	0.14	0.147	0.45	0.31	1.345	−0.023	−0.082	0.01	0	0.003	0	0.152	0.032
CD	0.079	0	0.11	0.03	−0.095	−0.02	0.675	0.077	−0.03	0.065	0	−0.005	−0.054	0.112
NSW	0.154	−0.041	−0.025	0.05	0.018	−0.08	0.077	0.821	0.142	0.102	−0.027	0	−0.099	−0.002
Covid B	−0.061	0.037	0.126	0.05	0.041	0.01	−0.03	0.142	0.835	0.162	0.017	0.023	−0.136	0.002
Covid F	−0.031	−0.024	0.007	0.02	0.01	0	0.065	0.102	0.162	0.57	0	0.065	0.058	0.028
Covid D	0	1.655*e*−4	0	0	0.003	0.003	0	−0.027	0.017	0	1.035	0.97	0.014	0
Covid Tot	−0.011	0	−0.009	−0.01	3.443*e*−4	0	−0.005	0	0.023	0.065	0.97	1.097	0	−0.004
Gender	−0.009	−0.009	0.009	0.15	0.045	0.152	−0.054	−0.099	−0.136	0.058	0.014	0	0.794	0.064
CPG	0.043	0.048	0.2	0.1	0.106	0.032	0.112	−0.002	0.002	0.028	0	−0.004	0.064	0.736

*The diagonal reports the Strength centrality index, which is the sum of all the weights that a node receives. Strength also measures the predictability of a node given the others*.

We wish to pinpoint here a few critical observations. If we put the focus on the beliefs of having had the Covid-19, we can see that it is associated with the conviction that also a close relative number had it. Interestingly, it is also associated with gender. About gender interpretation, 0 implies that the gender of the participant was not related to the other node under investigation. When an edge was different from 0, means that the gender was associated. Specifically, gender was coded as follow: 0 = male; 1 = female. By adopting this code, a positive edge indicates that women had a positive association with the associated node. A negative edge indicates that men are positively associated. It is then related to the work in presence. Moreover, it is also associated with the quality of sleep, the perceived stress and the somatization. Notably, it is only marginally associated with anxiety state, assessed through the BAI, and with the grade of chronic pain. The contextual level (i.e., number of positive cases in the Region, number of deaths in the Region) is only marginally associated with the belief of having had the Covid-19.

By focusing the observation on the belief that a family member had the Covid-19, we can observe that it is associated with the total number of cases recorded in the Region, suggesting a stronger influence of contextual factors, although the number of deaths is not predictive. Again, a strong predictor is to work in presence that seems the biggest predictor of the beliefs of Covid-19 infection. Interestingly any of the stress, anxiety, or somatization variables are predictive of thinking that the family member has had the Covid-19.

### Network B

**Figure 2:**
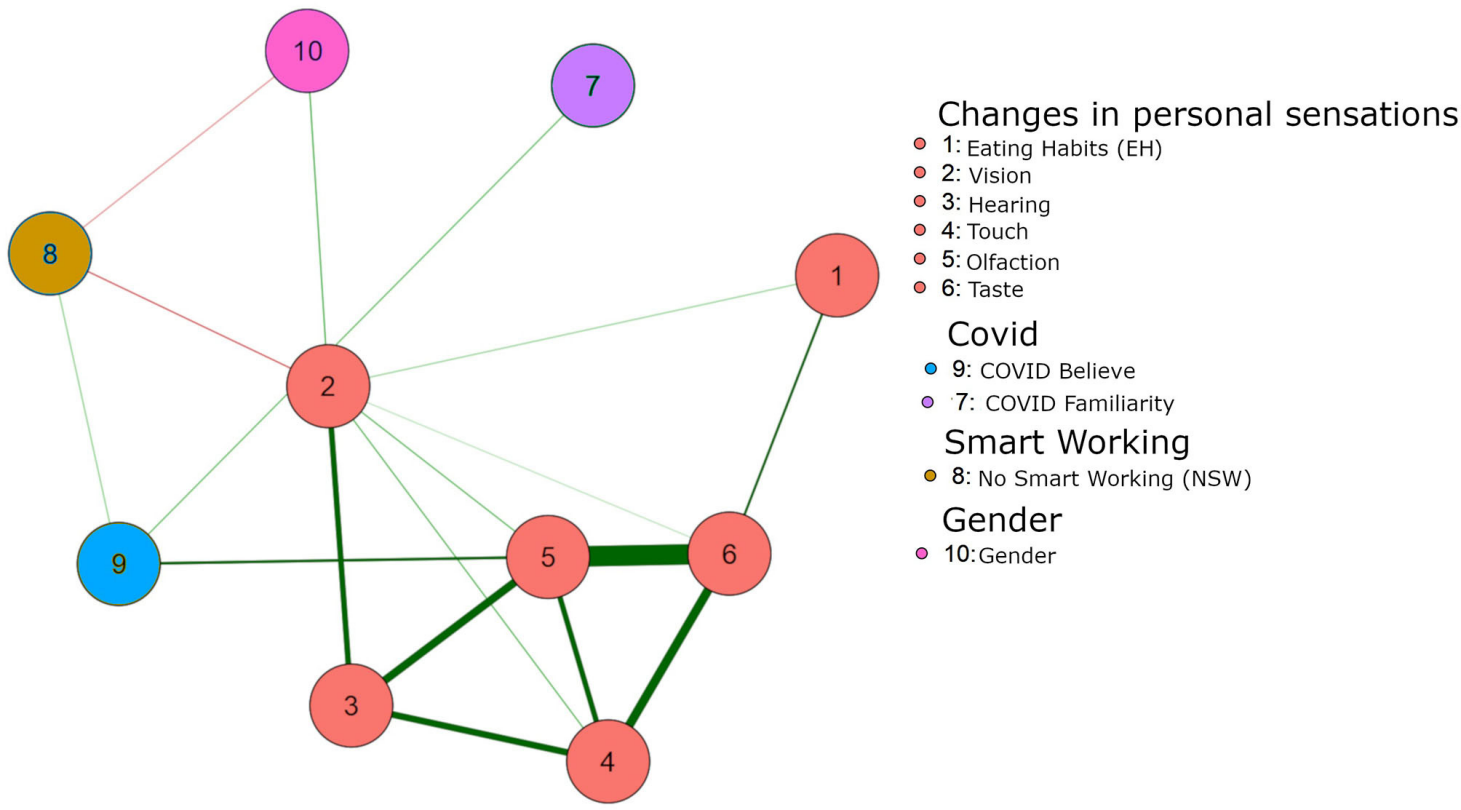
Green lines indicate positive associations. Red lines indicate negative associations. Edges represent the direct association between two edges. The nodes indicate the variables as follow: 1 Eating Habits (EH); 2 Vision; 3 Hearing; 4 Touch; 5 Olfaction; 6 Taste; 7 Covid F; 8 NSW; 9 Covid B; 10 Gender.

Figure 2 represents the best network estimated from the data. The exact value of all edges is reported in Table 2, the strength centrality index is reported on the diagonal of the same table.

**Table 2:** The matrix reports the network weights, which corresponds to a regularized odd ratio.

**Variable**	**EH (0 = no)**	**Vision**	**Hearing**	**Touch**	**Olfaction**	**Taste**	**Covid F**	**NSW**	**Covid B**	**Gender**
EH (0 = no)	−0.84	0.269	0	0	0	0.846	0	0	0	0
Vision	0.269	0.202	1.274	0.473	0.463	0.167	0	−0.496	0	0.536
Hearing	0	1.274	0.447	1.414	1.593	0	0	0	0	0
Touch	0	0.473	1.414	0.673	1.242	1.708	0	0	0	0
Olfaction	0	0.463	1.593	1.242	1.71	3.188	0	0	0.902	0
Taste	0.846	0.167	0	1.708	3.188	1.238	0	0	0.317	0
Covid F	0	0	0	0	0	0	−1.116	0	0.435	0
NSW	0	−0.496	0	0	0	0	0	−0.854	0.298	−0.285
Covid B	0	0	0	0	0.902	0.317	0.435	0.298	−0.5	0
Gender	0	0.536	0	0	0	0	0	−0.285	0	−0.959

*The diagonal reports the Strength centrality index, which is the sum of all the weights that a node receives. Strength also measures the predictability of a node given the others*.

A key observation is that the belief of having had the Covid-19 is directly related to the self-perception of changes in olfactory system. The other senses are not predictive *per se*. This is true still in the presence of the social predictor of having a job that requires to work in presence. Notably, the beliefs that a family member had Covid-19 is not related to any changes of perception in oneself. While this is of course intuitive (“my sensory changes do not predict the probability that you had the Covid-19”), it works somehow as a control result within the model, suggesting the validity of the estimated edges. An additional remark can be done on the relations that sensory changes have between themselves that suggests that they go hand-in-hand, so that a change in one sense is predictive of any other sense, although only olfactory perception is related to the Covid-19.

## Discussion

The empirical study of the consequences of Coronavirus on the mental health of the world population is attracting the interest of numerous national and international institutions. Several studies investigated the risks for the psychological well-being of individuals in quarantine, the main responses to stress (18), the risk perception (53, 54), the individual emotion (55), and the social behaviors related to the current pandemic and related restrictive measures. In the field of secondary prevention, the collected data may prove useful prospectively to structure *ad-hoc* interventions, aimed at enhancing the adaptation of individuals, improving the quality of life after the emergency and reducing the psychological symptoms deriving from exposure to stress (e.g., anxious, phobic, depressive symptoms, post-traumatic response) (56, 57).

In the field of health sciences, understanding the consequences on mental health in the time of the Coronavirus is becoming an increasingly urgent aspect, which must be contextualized with current events (58). How can we return to a psychosocial adjustment equal to the previous one without an understanding of the psychosocial impact of the crisis? To date, looking at the evidence of studies carried out in China, probably, can give us a chronological advantage in understanding the phenomenon.

Another open question concerns the need to find strategies for managing patients. For example, there are studies on the effects of lockdown in neurological patients, in neurodegenerative disorders (59), in Alzheimer's disease (60), in migraine and headache (61). These studies show us how the symptoms vary according to this new clinical/social situation and how remodeling is necessary to address the request for therapy and the therapeutic offer in a new way.

The Coronavirus and the consequent lockdown have made it so that interest in the importance of psychological well-being re-emerges and, starting from this situation of discomfort, we can only hope that psychological health is the ultimate and definitive goal of a future dedicated to the promotion of human well-being. The data from our study focuses on relevant connections. The first evidence is gender related. There are variations in the responses related to gender aspects, where, for example, women who are in a workplace situation present higher levels of perceptive stress and higher levels of psychophysiological symptoms (i.e., sleep quality, perceived stress, and somatization). These variables seem to be little associated with anxiety levels, which instead are more connected with chronic pain. This result is not in line with literature findings, that described women as more resilient than men during the lockdown (23). We can hypothesize that, given that this greater susceptibility of women is connected to the working condition in presence, probably this greater difficulty is due to seeking a mediation between a family management condition that is not compatible with work in presence.

A particular result is related to the perception of having had Covid-19. This “belief” is connected in an extremely marginal way with the number of positive cases and the number of deaths in the region. Probably this result can be connected to the fact that the perception of the regional situation is only indirectly perceptible. Conversely, the physical symptom connected to changes in breath, in temperature, in psychophysiological aspects such as those assessed (e.g., somatization and perceived stress), can be strongly linked to the idea of being sick, especially in a period in which all media attention is focused on flu symptoms, however frequent and common in the population and not necessarily caused by Covid-19.

Instead, the focus to the belief that a familiar had the Covid-19, is associated with the total number of cases recorded in the Region, suggesting a stronger influence of contextual factors, although the number of deaths is not predictive. This aspect can be due to the different levels between the number of death caused by Covid-19 (in any case passed by the media as “national” and rarely “regional” numbers) and the symptoms experimented within a familiar environment. Interestingly, the perceived stress, the anxiety level, or the somatization is not predictive of thinking that a family member suffered of Covid-19.

Another strong predictor seems to be the work in presence, that is the best predictor of the belief of suffering from Covid-19 infection. This could be motivated by direct contact with several people and by the awareness that the preventive measures used (masks and hand disinfection) are often not so efficient at a preventive level (see the large number of doctors and nurses that were infected in health facilities, where prevention devices are mandatory) (62).

Pointing attention to the perception, a key observation is that the beliefs of having had the Covid-19 is directly related to the self-perception of changes only in olfactory perception and indirectly to gustatory perception and not in another sensorial/perceptive modality. According to our model the gustatory modification could be directly related to the olfactory modification (and not directly related to Covid-19). This is true still in both the social predictors linked to the work (i.e., smart working, work in presence). Notably, the beliefs that a family member got the infection are not related to any changes of perception in oneself. Another interesting relation is the additional remark that sensory modalities can vary together, so that a change in one sensory modality can be predictive of any other sense, although only the olfactory is related to the Covid-19.

We can suppose that this happens for two reasons: both because the information on the Covid-related olfactory symptoms is immediately and massively passed on by the media, and because the sense of smell is strongly modulated by stressful perception (63).

The limitations of the study are strongly connected to the lack of data related to the model at an early stage, in the absence of the pandemic situation. Precisely for this reason, an exploratory model was proposed. In fact, to evaluate the long-term psychophysiological effects due to the pandemic condition, we should evaluate the same proposed model with follow-up.

Other limits are also connected to the correct interpretation of the results.

First, although the sample size is quite large, including people from diverse regions of Italy and is wider than the typical samples of psychology studies, it is important to remember that it cannot be considered representative of the Italian population. Indeed, the sampling strategies available for the study, and adopted, restricted the generalizability of the results.

Second, the edges reflect the unique associations left after conditioning on all the other variables. Thus, it is the association between two variables net of the other variables studied.

It is possible that an unmeasured variable mediates the relation between two edges. This might be unimportant, as it is a feature of any kind of measurement and analysis, but it is important to remind that an association is such only considering the other variables of the network.

Third, all the variables are self-reported, possible biases affecting every self-report measure can potentially affect also our results. For example, sensory changes are not objectively proved, but they are the self-perception of a change. This is not problematic *per se*, but it is a necessary reminder for the proper interpretation of the results.

Nonetheless this, we can conclude that the beliefs of having had the Covid-19 could be related to individual variables, while the familiarity of Covid-19 disease could be linked to contextual factors. Moreover, the self-perception of perceptive modulations, showed that olfactory variations were related to the belief of having had the Covid-19, and that gustatory perception is strictly linked to olfactory one. This point is strictly relevant: by one side because the communication that olfactory impairment is one of the most easy to read Covid-19 symptoms, by the other side why olfactory is one of the most stress-impaired sense (probably this is due to cortical/anatomical olfactory pathways, strictly linked with limbic system). Finally, as also showed in some previous reports, sleep impairment appears to be very relevant in the experience of Covid-19 infection.

This Italian photograph of a particular social-health moment, although it describes, in an exploratory way, the major stress related psychophysiological responses. Furthermore, it allows us to understand how, an altered and not predictable “ecological” system, our psychophysiological responses can be related to cognitive aspects. In this case, the management of the stressful representation is fundamental and is certainly mediated by communication systems. A communication that allows the correct evaluation of events and the correct value of prevention would probably allow a better management of the stressful representations. The connection between communication aspects, stressful representation and psychophysiological variables could also be investigated in a future study.

## Data Availability Statement

The original contributions generated in the study are included in the article/supplementary material, further inquiries can be directed to the corresponding author/s.

## Ethics Statement

The studies involving human participants were reviewed and approved by Ethical approval was obtained from the local Ethics Committee of the University of Torino (prot. n. 147807, 30.03.2020). The patients/participants provided their written informed consent to participate in this study.

## Author Contributions

SI conception and design of the study, descriptive data analysis, drafting of the manuscript, survey dissemination, and data interpretation. DR data analysis, data interpretation, drafting the manuscript. FG design of the study and drafting of the manuscript. VB, CU, GC, MP, GK, VB, MF, MT, and MV drafting of the manuscript, contribution to the design of the study and survey dissemination. AG survey editing and dissemination. ER contribution to the design of the study, survey dissemination and manuscript revision. All authors contributed to the article and approved the submitted version.

## Conflict of Interest

The authors declare that the research was conducted in the absence of any commercial or financial relationships that could be construed as a potential conflict of interest.
