# Corrigendum: Major Stress-Related Symptoms During the Lockdown: A Study by the Italian Society of Psychophysiology and Cognitive Neuroscience

**DOI:** 10.3389/fpubh.2021.711132

**Published:** 2021-06-11

**Authors:** Sara Invitto, Daniele Romano, Francesca Garbarini, Valentina Bruno, Cosimo Urgesi, Giuseppe Curcio, Alberto Grasso, Maria Concetta Pellicciari, Giacomo Koch, Viviana Betti, Mirta Fiorio, Emiliano Ricciardi, Marina de Tommaso, Massimiliano Valeriani

**Affiliations:** ^1^INSPIRE LAB - Laboratory of Cognitive and Psychophysiological Olfactory Processes, Department of Biological and Environmental Sciences and Technologies, University of Salento, Lecce, Italy; ^2^Department of Psychology, University of Milano-Bicocca, Milan, Italy; ^3^Manibus Lab, Department of Psychology, University of Torino, Torino, Italy; ^4^Department of Languages and Literatures, Communication, Education, and Society, University of Udine, Udine, Italy; ^5^Dipartimento di Scienze Cliniche Applicate e Biotecnologiche, Università Degli Studi Dell'Aquila, L'Aquila, Italy; ^6^UniCamillus, Saint Camillus International University of Health Science, Rome, Italy; ^7^Fondazione Santa Lucia IRCCS, Rome, Italy; ^8^Dipartimento di Neuroscienze e Riabilitazione, Università di Ferrara, Ferrara, Italy; ^9^Department of Psychology, Sapienza University of Rome, Rome, Italy; ^10^Department of Neurosciences, Biomedicine and Movement Sciences, University of Verona, Verona, Italy; ^11^IMT School for Advanced Studies, Lucca, Italy; ^12^Department of Basic Medical Science, Neuroscience and Sense Organs, University of Bari Aldo Moro, Bari, Italy; ^13^Neurology Unit, Bambino Gesù Hospital, Rome, Italy

**Keywords:** Covid-19, pain, sleep habits, olfactory perception, lockdown, stress, anxiety symptoms

An author name was incorrectly spelled as **Giacomo Kock**. The correct spelling is **Giacomo Koch**.

In the published article, there was an error in affiliation **7**. Instead of “Dipartimento di Neuroscienze, Policlinico Tor Vergata, Rome, Italy,” it should be “**Fondazione Santa Lucia IRCCS Rome, Italy and Dipartimento di Neuroscienze e Riabilitazione, Università di Ferrara, Ferrara, Italy**”.

In the published article, there was an error regarding the affiliation**(s)** for **Giacomo Koch**. As well as having affiliation(s) **7**, they should also have affiliation **8**.

The authors apologize for this error and state that this does not change the scientific conclusions of the article in any way. The original article has been updated.

